# Hepatitis C Virus and Hepatocellular Carcinoma: Pathogenetic Mechanisms and Impact of Direct-Acting Antivirals

**DOI:** 10.2174/1874357901812010016

**Published:** 2018-02-28

**Authors:** Ivan Schietroma, Giuseppe Corano Scheri, Claudia Pinacchio, Maura Statzu, Arnolfo Petruzziello, Vincenzo Vullo

**Affiliations:** 1 Department of Public Health and Infectious Diseases, “Sapienza” University of Rome, Rome, Italy; 2 Department of Molecular Medicine, Laboratory of Virology, “Sapienza” University of Rome, Rome, Italy; 3 Virology and Molecular Biology Unit, Department of Diagnostic Pathology, Istituto Nazionale Tumori, IRCCS Fondazione G. Pascale, Naples, Italy

**Keywords:** HCV, HCC, DAA’s, Immunity, Viral factors, Chronic infection

## Abstract

**Introduction:**

Globally, between 64 and 103 million people are chronically infected with Hepatitis C virus (HCV), with more than 4.6 million people in the United States and is associated with more than 15.000 deaths annually. Chronic infection can result in cirrhosis and hepatocellular carcinoma.

**Explanation:**

Epidemiological studies have indicated that persistent infection with hepatitis C virus (HCV) is a major risk for the development of hepatocellular carcinoma (HCC), mainly through chronic inflammation, cell deaths, and proliferation. Despite the new direct-acting antiviral drugs (DAA’s) being able to clear the HCV, HCC recurrence rate in these patients is still observed.

**Conclusion:**

In this review we highlighted some aspects that could be involved in the onset of HCV-induced HCC such as immune system, viral factors and host genetics factors.

Moreover, we focused on some of the last reports about the effects of DAA’s on the HCV clearance and their potential implications in HCC recurrence.

## 1 INTRODUCTION

Hepatocellular carcinoma (HCC), also known as malignant hepatoma, is a primary malignant tumor of the liver arising from the liver cells (hepatocytes) [1].

Amongst the most common tumors worldwide [2], it occurs predominantly in patients with underlying chronic liver disease and cirrhosis.

In the United States, HCC is the ninth leading cause of cancer deaths [3]. Despite advances in prevention techniques, screening, and new technologies in both diagnosis and treatment, incidence and mortality continue to rise (662,000 deaths worldwide per year) [2].

HCC occurs more often in males than females (ratio 2,4:1) and it is common between the age of 30 to 50 [2], peaking at approximately 70 years of age and with a higher incidence in Eastern and Southern Asia, Middle and Western Africa, Melanesia, and Micronesia/Polynesia [3].

### 1.1 Risk Factors

The distribution of risk factors in patients with HCC depends on the ethnic group, race or geographical region. The most common risk factors are: infection with Hepatitis B virus (HBV) or Hepatitis C virus (HCV), alcoholic liver diseases, Non-Alcoholic Fatty Liver (NAFLD), Non-Alcoholic Steatohepatitis (NASH) and aflatoxins [1]. Less common causes include sex and metabolic and genetic diseases such as diabetes mellitus, obesity, hemochromatosis and Wilson’s disease.

The above-mentioned risk factors often lead to the formation and progression of cirrhosis, which is present in 80 to 90% of patients with hepatocellular carcinoma.

In the developing world, viral hepatitis (primarily hepatitis B) continues to represent a major risk for the development of HCC. Worldwide, chronic HBV infection accounts for approximately 50% of all cases of hepatocellular carcinoma and virtually all childhood cases.

The World Health Organization estimates that every year 3–4 million people get infected with hepatitis C virus (HCV) worldwide. Whereas 130–170 millions of chronically infected individuals are at risk of developing liver Cirrhosis, Hepatocellular Carcinoma (HCC), or both. These infections induce over 350 thousand deaths per annum, but the manifestation varies widely with geographical deviations. The prevalence is as low as 0.1–1% in northern Europe to 2.5–3.5% in southern Europe. Amongst the most affected countries is Egypt, with 22% of its population infected, followed by Pakistan and China, with more than 3% of their population infected with HCV [4].

Hepatitis C virus infection was first suspected in the 1970s, when most blood transfusion infections were associated with either hepatitis A or hepatitis B virus. This new type of blood-transmitted hepatitis was then called “non-A, non-B” hepatitis.

The genome of HCV was identified in 1989, and the name hepatitis C was subsequently applied to human infection caused by this single-strand Ribonucleic Acid (RNA) virus of positive polarity [5].

Although its origin remains unclear, HCV might have originated from zoonotic sources such as non-human primates (*e.g*. monkeys, apes) and mammals (*e.g*. dogs, horses) [6].

Hepatitis C virus belongs to the Hepacivirus genus, *Flaviviridae* family, and exhibits high genetic variability. There are seven different genotypes, and more than 70 subtypes [5] and their global prevalence is unequally distributed. Amongst 7 HCV genotypes, genotype 1 and 3 account respectively for 46.2 and 30.1% of the global infections; genotypes 2, 4, and 6 are present in approximately 22.8% of HCV infections and genotype 5 represents the remaining less than 1%; genotype 7 has been identified so far in very few patients originating from Central Africa [6].

A single HCV particle is approximately 68 nm in diameter [6].

A lipid bilayer envelope surrounds a capsid. The viral envelope bears E1 and E2 glycoprotein complex that plays a fundamental role in viral and host cell interaction [4].

Viral genome, comprised of 9600 nucleotides, encodes a 3000 aminoacid polyprotein, which is post-translationally modified into three structural proteins and six non-structural proteins. Structural proteins, Core, E1, and E2, create the capsid and the envelope, while non-structural proteins, NS2, NS3, NS4A, NS4B, NS5A, and NS5B, are the building blocks for the particle and play a vital role as genome replicating proteins [4].

Alcohol abuse is an important risk factor for development accounting for 40%–50% of all HCC cases in Europe. The relationship between alcohol and liver disease correlates with the amount of alcohol consumed over a lifetime, with heavy alcohol use rather than social drinking being the main risk of HCC.

Chronic diseases such as diabetes mellitus and obesity increase the risk of HCC. Diabetes mellitus directly affects the liver because of the essential role the liver plays in glucose metabolism.

Hyperinsulinemia has been associated with a threefold increased risk of HCC. It is believed that the pleotropic effects of insulin which regulate the anti-inflammatory cascade and other pathways inducing cellular proliferation, play a role in carcinogenesis.

It is well established that obesity is associated with many hepatobiliary diseases, including nonalcoholic fatty liver disease (NAFLD), steatosis, and cryptogenic cirrhosis, all of which can lead to HCC.

Gender may also play a role in the development (growth, evolution, production) of Hepatoma, occurring more often in males, with a ratio of 2:1– 4:1 . Males are more likely to get infected with viral hepatitis, consume greater quantities of alcohol, smoke cigarettes, and have a higher body mass index than women. Higher testosterone levels may also increase the incidence in males and the formation of liver adenomas.

Aflatoxins, metabolites of the fungi Aspergillus flavus and Aspergillus parasiticus are frequent contaminants of food, and are associated with a high rate of HCC development [7]. The risk of HCC with aflatoxin depends on the amount and duration of exposure. Aflatoxin exerts a synergistic effect on hepatitis B- and C-induced liver cancer, with the risk being 30 times greater with chronic hepatitis B plus aflatoxin exposure than with aflatoxin exposure alone [3, 8].

Metabolic and genetic diseases associated with HCC include hemochromatosis, Wilson’s disease, alfa-1 antitrypsin disease, tyrosinemia, glycogen-storage disease types I and II, and porphyrias. Other risk factors may include cigarette smoke, associated with a significant increase in the development of HCC [3].

### 1.2 Pathogenesis

As a blood-borne virus, HCV can be transmitted over blood transfusions, needle sharing, sexual contacts, or maternal transmission. Although HCV can circulate in many human organs, it especially infects hepatocytes in the liver and evades the host innate and adaptive immune system. With an incubation period of 2 to 12 weeks, HCV infection begins with an acute phase that usually goes undiagnosed, during which symptomatic infections (10% to 15%) and asymptomatic infections (85% to 90%) are observed. Approximately, 25% to 52% of symptomatic infections and 10% to 15% of asymptomatic infections undertake spontaneous viral clearance, indicating that HCV is cleared from HCV-infected patients by specific immune responses. If not cleared, acute HCV evolves into chronic HCV. Without proper treatment, patients with chronic HCV are threatened by serious complications such as cirrhosis, liver cancer, and liver failure. Liver damage is a consequence of long-lasting inflammation when host immune responses are activated to fight HCV infections [6].

Hepatocarcinogenesis is a highly complex multistep process: it starts from hepatic stem cells or mature hepatocytes under the condition of chronic liver disease initiated by oxidative stress, chronic inflammation and cell death followed by uncontrolled proliferation/restricted regeneration and permanent liver remodeling.

In response to cytokine stimulation caused by hepatocyte injury, extensive compensatory cell proliferation and regeneration take place, followed by fibrosis and cirrhosis, particularly driven by the synthesis of extracellular matrix components from hepatic stellate cells. Finally, in this carcinogenic environment, hyperplastic and dysplastic nodules form and normal liver tissue turns cancerous. The molecular pathogenesis of HCC involves different genetic/epigenetic aberrations and alterations in multiple signaling pathways leading to the known heterogeneity of the disease concerning its biologic and clinical behavior [1].

## 2 IMMUNITY

In patients persistently infected with HCV, chronic inflammation resulting from the immune response against infected hepatocytes typically lasts many decades and it frequently leads to progressive liver disease that ranges from inflammation to severe fibrosis, that can culminate in hepatic cirrhosis and Hepatocellular Carcinoma (HCC) [9].

A variety of PRRs (Pattern Recognition Receptors) sense viruses as foreign invaders within the host cell through specific PAMP (Pathogen Associated Molecular Patterns) recognition to activate innate immune signalling [10]. The 3 major classes of PRRs include Toll-like receptors (TLRs), RIG-1-like receptors (RLRs), and nucleotide oligomerization domain (NOD)-like receptors (NLRs) [11].

NS5B, the HCV RNA-dependent polymerase, can activate innate immune signalling through dsRNAs that are transcribed from nonspecific cellular templates. Both TLR3 and RIG-1 can trigger cellular responses to RNA produced by NS5B, but Vegna *et al*. showed that NS5B induces and activates NOD1, a receptor classically associated with a response to bacterial infection, that has as downstream effector RIPK2, which leads to MAPK activation, type I interferon production and NF-κB-dependent signalling [9]. The persistent inflammatory response observed in the liver of HCV-positive patients thus impacts many aspects of HCV-associated pathology, including HCC, by creating a favorable environment for disease progression [9].

Recently, Lopes *et al*. [12] have shown that TLRs are closely related to carcinogenesis. Also myeloid differentiation factor 88 (MyD88), the TLR and interleukin (IL)-1 receptor adaptor molecule, has been linked to tumorigenesis. Kinowaki *et al*. [13] examined the expression of MyD88 in human HCC tissues, finding that attenuated expression of MyD88 in HCC tissues is associated with tumor progression. This was in contrast with other recent reports [14-16].

Tumor necrosis factor-α-induced protein-8 like-2 (TIPE2) is a newly identified protein essential for the maintenance of immune homeostasis and for tumorigenesis suppression. Recently, a study showed that another HCV protein, NS5B, can lead to hepatocellular carcinogenesis by promoting TIPE2 degradation [17].

Golgi protein 73 (GP73) is a serum biomarker for liver disease and HCC with high specificity and sensitivity. However, the mechanism underlying GP73 regulating HCV infection is poorly understood. Zhang *et al*. [18] discovered that GP73 acts as a negative regulator of host innate immunity: it binds directly to mitochondrial antiviral signaling protein (MAVS) and TNF receptor-associated factor 6 (TRAF6) to promote their degradation, which result in the repression of host innate immunity and facilitation of HCV infection.

Persistent infection by hepatitis C virus (HCV) represents one of the main risk factors for HCC and has been shown to impair NK-cell response, mainly exerting a suppressive function that might play a role in tumor promotion or progression [19].

With the aim to investigate the phenotype and function of peripheral blood NK in HCV-linked HCC and their possible prognostic implications, Cariani *et al*. [19] evaluated a cohort of patients with HCV-related HCC: they found a decreased expression of inhibitory receptor NKG2A and of molecules linked to effector function (CD3z, perforin) in peripheral blood NK-cells from HCC patients. The study thus supports prognostic relevance of NK-cell phenotype and function in HCC.

## 3 HCV AND HCC: WHAT ABOUT GENETICS?

There are 3 IFN-lambda genes that encode 3 distinct but highly related proteins denoted as IFN-lambda1, -lambda2, and -lambda3. These proteins are also known as interleukin-29 (IL-29), IL-28A, and IL-28B, respectively. Only recently IFN lambda 4, a new member of the family, was discovered. These cytokines comprise the type III subset of IFNs [20]. In HCV infected patients, genetic variations in the IFNλ locus are associated with spontaneous viral clearance and type I IFN-based treatment success [21, 22]. Three major SNP near IFNL3 and INFL4 genes correlate with HCV treatment response: rs12979860(C/T), rs8099917(T/G) and rs368234815(TT/ΔG) [23]. The molecular mechanisms that control the association between IFNλ polymorphisms and the clinical outcome of HCV infection still remain unclear.

In 2015, a study demonstrated a correlation between the rs8099917 SNP and IL28B serum levels, while other SNPs, including rs12979860 failed to show a similar correlation. They observed that the T-allele of rs8099917 was correlated with reduced IL28B serum levels. Also, they found that IL28B serum levels had significant correlation with the different outcomes of HCV infection, especially among patients suffering from cirrhosis and HCC, where IL28 serum level tend to increase with the progression of the disease, but even if IL28B variants might play a significant role in HCV infection, they may not be considered as risk factors in the progression of the infection to advanced stages such as HCC. However, further investigations are needed to explain the effect of IL28B levels on disease progression [24].

TLRs are a group of molecules that are essential for the innate immune response against pathogens. De Re *et al*. in 2016 investigated the hypothesis of a functional connection between IL28B and toll-like receptor 2, a pattern recognition receptor that has specific polymorphism associated with hepatocarcinogenesis. This study emphasizes that both TLR2 and IL28B polymorphism may have a role in directing HCV-progression towards hepatic diseases: in particular, the presence of IL28B-C allele in homozygosity has a potential protective effect towards chronic infection and liver diseases, but the simultaneous presence of at least one TLR2-del allele abolished this effect; the TLR2-del/del state appeared also closely linked with HCC condition [25].

Lately, Yuan *et al*. described the TLR3 mediated antitumor activities towards HCC. In 2017 a study of Al-Anazi investigated the influence of genetic variants within TLR3 with the purpose to determine the association with HCV infection and HCV-related liver damage that results in cirrhosis and HCC. They found that rs78726532 was strongly associated with HCV infection. Moreover, the rs78726532 GG genotype had a protective role for HCV infection. Three more TLR3 SNPs were associated with HCV related end stage liver disease progression (liver cirrhosis and HCC) [26].

In 2014, Mc Farland *et al*. reported the identification of a functional polymorphism (rs4803217) located in the 3′ untranslated region (3′ UTR) of the IFNL3 mRNA that dictates transcript stability. This polymorphism influences AU-rich element-mediated decay as well as the binding of HCV-induced microRNAs during infection. Together, these pathways mediate robust repression of the unfavorable IFNL3 genotype [27]. More recently, another study compared rs4803217 to IFNL4-ΔG/TT, a functional variant that controls generation of the IFNL4 protein, showing evidence that the IFNL4- ΔG allele is a primary variant for impaired clearance of HCV [28].

Another study has recently shown the association between SNPs in 3 genes involved in early immune response against HCV and the risk of progressive liver disease: low molecular mass polypeptide 7 (LMP-7), interleukin 28B (IL28B) and 2’-5’oligoadenylate synthetase 1 (OAS1). In particular, SNPs in LMP-7 and IL28B rs12979860 are associated with the development of HCC [29].

These findings highlight the importance of host genetic factors in determining the early stages of HCV infection and the development of HCV-associated HCV diseases.

## 4 VIRAL AND NON-VIRAL FACTORS INVOLVED IN HCV-RELATED HCC

The escape from the immune system may represent one of the mechanisms by which HCV establishes persistent infection and which contributes to the maintenance and development of cancer. Epidemiological studies on the association between hepatitis C virus infection and Hepatocellular carcinoma (HCC) development have begun in rapid succession after the development of the first detection test specific antibody [30]. A direct role in inducing HCC was highlighted in transgenic mice for core expression in which the induction of hepatic carcinoma is accelerated [31]. Direct and indirect pathways of carcinogenesis are responsible for HCC development and understanding of these pathways will be key to devising new treatment strategies. The most important mechanisms are: direct pathways involving the HCV core protein, indirect injury from oxidative stress and steatosis leading to hepatocyte death, and micro RNA (mi-RNA) instability. The possible mechanisms for HCC induction by the HCV virus are, however, little known being an RNA virus in which the replicative cycle, which occurs in the cytoplasm, a DNA phase is not present or the possibility of viral integrals or viral oncogenes is known [32]. It is believed that an important role is given by viral persistence in immune stimulus incessant exercise. Consequently, the HCV core protein plays a pivotal role in the development of direct mechanisms of HCC; in fact, the core deregulates the pathways by inhibiting retinoblastoma protein (RB) and p53 tumor suppressor [33]. Furthermore, HCV NS5 inhibits the apoptosis regulators BCL-2, and it is the cause of abnormal activation of signaling pathways that promote growth, such as Wnt/beta catenin and mammalian target of rapamycin (mTOR) [34]. Further, hypotheses are related to a possible indirect role of some viral proteins, in particular core protein and NS5. HCV core protein interacts with different routes of intracellular signal transduction (TNF-α, NF-kB, activation of the path induced by oxidative stress: kinase MAPK, JNK / SAPK); inhibits and induces apoptosis Fas- and TNF-α mediated thus contributing to increased survival or increased proliferation of hepatocytes [35]. The research group of Dr. Conti highlighted, during HCV life cycle with the help of a HCV infection model, a functional interaction between Small Heterodimer Protein 1 (SHP1), a steatogenic protein, and HCV NS5A protein. They demonstrated that SHP1 silencing (siSHP1) reversed the pro-oncogenic effects of HCV infection, inducing a significant decrease in liver lipid accumulation and in NS5A protein expression. Moreover, siSHP1 causes a strong modulation of some genes involved in HCV-related EMT, such as: HNF4, a central regulators of hepatocyte differentiation, E-Cadherin, SNAILs. This data suggest that SHP1 has been shown not only to be strictly connected to the pathogenesis of HCV-related liver steatosis, but also to its progression towards liver transformation [36]. HCC microarray analysis suggested that some clusters of genes involved in growth cells are over-regulated (PCNA, members of the cycling family: CDC20, CDK4, Myb) while other genes of the Wnt catenin-β family and metalloproteinases were modulated in the expression of HCC [37].

The expression of miRNAs in hepatic tissue is related to the pathogenesis of liver disease, including, hepatocellular carcinoma. Different mi-RNA are downregulated in HCC and need more studies to evaluate the real role of this RNA [38-40]. These and other papers indicate that miRNAs may be biomarkers for the evaluation of HCC, but candidate miRNAs are different. Further research is definitely required in future works.

## 5 HCV AND HCC: ANY RELATIONSHIP WITH DAAS?

The advent of novel direct-acting antivirals (DAAs) has totally changed the landscape of HCV therapy. In fact, HCV infection cure rate is estimated at around 90%, with a sustained virological response (SVR) of around 95-97% [41, 42]. These results have raised the hope of an improvement in disease severity. One of the main expectations following DAA treatment is not only to eradicate the virus, but to improve liver function too, such as cirrhosis, fibrosis and HCC. In particular, the reduction of inflammation can lead to a decrease of cirrhosis and HCC.

Indeed, in the past, Interferon (IFN) based therapies have demonstrated that this treatment resulted in an increased HCV clearance and that SVR had reduced liver fibrosis, cirrhosis and HCC [43, 44]. These data are even more consistent considering the anti-tumor and anti-proliferative effects of IFN [45]. Nevertheless, given the adverse effects of IFN and ribavirin, a new class of direct-acting antivirals drugs has been introduced. DAAs are antiviral drugs that impact HCV replication at different stages, such as protein assembly or polymerase activity.

But what’s the real effect of DAAs on HCC in HCV populations? Not much data on the role of DAAs-induced HCV eradication in HCC occurrence are available. Recently, some controversial data have been published on the relationship between HCC recurrence and DAAs treatment in HCV spanish population. In fact, Reig *et al* show that despite a high rate of SVR after treatment with DAAs, an elevated number of HCC recurrence has been observed in their cohort. In particular, they included 55 HCV patients, with a complete radiological response after a prior history of HCC who received DAAs, as a final study population and they observed a radiologic tumor recurrence of (27.6%) (16 of 55 patients) [46]. The observed a very close time between DAAs treatment and cancer recurrence (median time 3,5 months). In their opinion, these data raise a concern about the benefit of DAAs on HCV patients with a prior HCC history.

Another study of Conti *et al*. found that, during the 24 weeks follow-up evaluation, HCC was detected in 26 of 344 HCV patients (7.6%). In particular, HCC developed in 17 of the 59 patients with a prior history of HCC (28.81%), and in 9 of the 285 (3.2%) who did not expertise cancer in their life [47]. In both these studies a high rate of SVR is observed, confirming real effectiveness of DAAs against HCV infection. When Conti *et al*. compared these data with HCC recurrence in HCV untreated population, they found a similar rate of cancer development, suggesting that it is not possible to affirm that DAAs treatment is involved in HCC.

Another study by The ANRS collaborative study group on hepatocellular carcinoma, in France, reported that no increased risk of HCC recurrence in HCV populations has been observed following DAAs treatment [48].

Furthermore, the HCC recurrence found by Conti *et al*. is similar to that found in an Italian study in which the authors indicate that HCC recurrence after 1 year from the surgical resection of the tumor is around 20% [49].

One of the most important concerns is to understand why a sudden HCC recurrence after DAAs treatment is observed.

An explanation of these results is not yet reached but one of the main hypotheses is that following DAAs treatment a perturbation of the immune system occurs.

In fact, it’s been hypothesized that changes in pro and anti-tumor signals after DAAs treatment could impact on the HCC recurrence. For example, DAAs therapy-induced SVR could cause the reduction of Natural Killer (NK) cells and their role in immuno surveillance, enhancing the growth of the undetected tumor [50]. This phenomenon is unlikely in HCV patients treated with IFN, because of the immuno modulatory and anti-proliferative effect of this cytokine [51].

Consistent with this hypothesis, an Italian study speculated that drugs could induce HCC recurrence through an increase in tumor cells dissemination. In particular, Villani *et al*. found an increase in vascular endothelial growth factor (VEGF) levels during DAAs therapy, while they normalized within 12 weeks at the end of therapy [52]. The serum of these patients, also, had angiogenic properties, when tested in an in vitro cells stimulation assay [52]. VEGF is a critical factor in angiogenesis, that is mainly responsible for tumor dissemination and it seems to correlate with a rapid development and recurrence of HCC [53]. The effects of DAAs on inflammatory pattern and on the immune system seem to be related to the onset of HCC in HCV population. Other studies reported changes in the immune system and the reduction of IFN producing cells following DAAs treatment [50, 54], suggesting that DAAs can reset HCV viral load but could be involved in modifying the inflammatory pattern and the immuno surveillance against HCC.

## 6 DAA-RAMS OCCURRENCE AND HCC DEVELOPMENT

An important issue to face is the onset of DAAs related- resistance associated mutations (RAMs). Treatment with DAAs that target the HCV protease NS3/4A, NS5A and the NS5B polymerase proteins can lead to more than 90% SVR [55].

HCV has higher diversity and variability in its genome compared to other viruses. During its replicative cycle, a lot of mutations occurs in the genome, creating a pool of virus variants termed “quasispecies” [56].

RAMs are often used to describe the amino acid substitutions that reduce the susceptibility of a virus to a drug.

Viral resistance after DAAs administrations occurs when some viral variants with reduced susceptibility to drugs are selected and replicate [57]. Resistance of HCV to DAAs is determined by many factors such as the genetic factors, related to the number and type of nucleotide substitutions and the number of mutations required for a virus to acquire full resistance to the drug [56]. Another factor involved in the resistance to DAAs is determined by the fitness of resistant virus populations, which is independent of the level of resistance conferred by RAMs [58].

Once resistance is acquired, a high risk of treatment failure occurs.

Sometimes, in patients with suboptimal response to treatment, HCV variants with a different level of susceptibility may exist naturally at low levels in the absence of drug, promoting resistance to direct acting antiviral agents and treatment failure [59].

Relapse of HCV in patients who initially respond to DAA may be due to the replication of a residual variant that remained below the limit of detection at the end of treatment. After relapse, viral sequencing may identify the virus sequence present after treatment, but sometimes an important phenomenon could occur after relapse; in fact it is possible that a viral population could evolve to wild type prior to sequencing after relapse, as it is not under selective pressure at the time [56].

The viral variants following a treatment failure are resistant to one or several of the drugs administered [56].

In these not rare cases the viral trigger is even more difficult to suppress and it could play a pivotal role in the onset of HCC. Indeed HCC risk was increased 17-fold in HCV-infected patients compared with HCV-negative controls and in many of these cases, HCC patients might have had HCV detected by polymerase chain reaction testing of liver tissue and/or serum, even if antibody to HCV (anti-HCV) was nondetectable [5]. A failure or relapse in HCV therapy may have a decisive weight in favoring the development of HCC. To ensure effective treatment the HCV subtype should be determined prior to therapy and resistance testing could be an important weapon to fight all the detrimental effects generated by virus escape and replication, which are the basis of HCC onset in HCV patients.

## CONCLUSION

Future studies on this phenomenon should report data from bigger sample size with a longer follow-up period. Moreover, the distinct clinical characteristics of the patients, included in the studies should be uniform. For example, Villani *et al*. show that Reig *et al*. recruited some patients who were treated with “non-curative” practice, such as chemoembolization, characterized by early recurrence tumor rates. This could explain the high rate of HCC recurrence observed by the Spanish group [52].

All these results show that, in particular for those patients who experienced HCC, all patients should be followed, both during and after DAAs therapy.

In conclusion, all data available do not seem to directly correlate DAAs therapy with HCC development in HCV treated patients, although patients who experienced HCC showed a high rate of tumor recurrence.
